# Antioxidant Activity of *Aspergillus fumigatus*

**DOI:** 10.5402/2011/619395

**Published:** 2011-04-11

**Authors:** Daljit Singh Arora, Priyanka Chandra

**Affiliations:** Microbial Technology Laboratory, Department of Microbiology, Guru Nanak Dev University, Amritsar 143005, India

## Abstract

The antioxidant activity of *Aspergillus fumigatus* was assayed by different procedures and correlated with its extracellular total phenolic contents. Different physio-chemical parameters were optimized to enhance the activity. The culture grown under stationary conditions for 10 days at 25°C at pH 7 gave the best antioxidant activity. Statistical approaches demonstrated sucrose and NaNO_3_ to be the most suitable carbon and nitrogen sources, respectively. Response surface analysis showed 5% sucrose, 0.05% NaNO_3_, and incubation temperature of 35°C to be the optimal conditions for best expression of antioxidant activity. Under these conditions, the antioxidant potential assayed through different procedures was 89.8%, 70.1%, and 70.2% scavenging effect for DPPH radical, ferrous ion and nitric oxide ion, respectively. The reducing power showed an absorbance of 1.0 and FRAP assay revealed the activity of 60.5%. Extracellular total phenolic content and antioxidant activity as assayed by different procedures positively correlated.

## 1. Introduction

Lipid peroxidation is a complex process occurring in aerobic cells and reflects the interaction between molecular oxygen and polyunsaturated fatty acids. Free radicals are known to take part in lipid peroxidation, which causes food deterioration, aging, and cancer promotion. Reactive oxygen species are also reported to be involved in asthma, inflammation, arthritis, neurodegeneration, Parkinson disease, vascular cardiac diseases, and diabetes [1]. Antioxidants act as radical-scavengers, and inhibit lipid peroxidation and other free radical-mediated processes; therefore, these are able to protect the human body from several diseases attributed to the reactions of radicals. Use of synthetic antioxidants to prevent free radical damage has been reported to involve toxic side effects thus necessitating the search for natural antioxidants and free radical scavengers [2].

In the past few years, natural antioxidants have generated considerable interest in preventive medicine and in the food industry. For the replacement of conventional synthetic antioxidants in food by natural products, medicinal plant extracts, spices, and mushrooms are considered to be a promising source [3]. Within these compounds, flavonoids and phenolic acids, phytochemicals with a large distribution in nature have been the object of a great number of studies of their antioxidative activity, which is mainly due to their capacity to act as free radical scavengers and/or as metal chelators [4]. In consequence, attention has been focused on the characterization of the antioxidant properties of products from several natural resources and isolation and identification of the constituents responsible for such activities.

Recently, fungi have emerged as the new sources of antioxidants in the form of their secondary metabolites [5, 6]. Fungi are remarkably a diverse group including approximately 1.5 million species, which can potentially provide a wide variety of metabolites such as alkaloids, benzoquinones, flavanoids, phenols, steroids, terpenoids, tetralones, and xanthones [7]. They demonstrate variety of bioactivities along with antioxidant properties and function as varied as their structure. They are exploited in medicine and industry and considered to be potential sources of new therapeutic agents.

Some fungi were isolated from soil and screened for antioxidant activity [8] and one of the best soil isolates (*Aspergillus fumigatus*) was selected for further study. Numerous techniques are available to evaluate the antioxidant activities of a compound, and just one procedure cannot identify all possible mechanisms characterizing an antioxidant. The comprehensive evaluation using different tests has been important in assessing the antioxidant activity. Therefore, different complementary test systems such as 1,1-diphenyl-2-picryl hydrazyl free radical (DPPH) assay, reducing power, ferrous ion and nitric oxide ion scavenging activity, and ferric reducing antioxidant power (FRAP) assay were used to assess the antioxidant potential of *A*.* fumigatus*. Different statistical designs (one-factor-at-a-time classical approach, Plackett-Burman design and response surface methodology) were used to enhance its activity. An effort has been made to work out the correlation (if any) between antioxidant activity and extracellular total phenolic content. 

## 2. Materials and Methods

### 2.1. Growth of *Aspergillus fumigatus*

*Aspergillus fumigatus *was isolated from soil of Attari area, Amritsar, Punjab, India (31° 37′ 59′′ North, 74°  51′ 56′′ East) and identified on the basis of standard protocol and the identity was confirmed by National Fungal Culture Collection of India, Agharkar Research Institute, Pune, India. To study the antioxidant potential, the fungus was grown on 50 mL Czapek dox's broth (sucrose 3%, NaNO_3_ 0.2%, K_2_HPO_4_ 0.1%, MgSO_4_ 0.05%, KCl 0.05%, FeSO_4_ 0.001%). The medium was inoculated with two discs (8 mm) of fungal mycelia obtained from 6-7 days grown culture on yeast extract glucose agar plates. The growth was carried out under stationary conditions at 25°C. After incubation of 10 days, the culture broth was centrifuged at 10,000 RPM at 4°C for 10 minutes and filtered through Whatman filter paper number 1 and the filtrate so obtained was used for further analysis. 

### 2.2. Assay Procedures for Antioxidant Activity

#### 2.2.1. 1,1-Diphenyl-2-picryl hydrazyl (DPPH) Free Radicals Scavenging Assay

The scavenging activity for DPPH free radicals was measured according to Zhao et al. [9] with slight modifications. To 2 mL of distilled water, 1 mL of 0.1 mM DPPH solution in ethanol and 0.5 mL of extract was added. The mixture was shaken vigorously and allowed to reach a steady state for 30 min at room temperature. Decolourization of DPPH was determined by measuring the decrease in absorbance at 517 nm, and the DPPH radical scavenging effect was calculated according to the following equation:

(1) $$\text{\%}\;\text{scavenging}\;\text{rate}=\left[1-\frac{\left(A1-A2\right)}{A0}\;\right]\times 100,$$

where *A*0 represents the absorbance of the control (DPPH without extract), *A*1 represents the absorbance of the reaction mixture, and *A*2 represents the absorbance without DPPH (DPPH was replaced by the same volume of distilled water). 

#### 2.2.2. Determination of Antioxidant Activity by Reducing Power Measurement

The reducing power of the extract was determined according to Chang et al. [10] with slight modifications. An aliquot of 0.5 mL extract was added to 0.1 mL of 1% potassium ferricyanide. After incubating the mixture at 50°C for 30 min, during which ferricyanide was reduced to ferrocyanide, it was supplemented with 0.1 mL of 1% trichloroacetic acid and 0.1% FeCl_3_ and left for 20 min. Absorbance was read at 700 nm to determine the amount of ferric ferrocyanide (Prussian blue) formed. Higher absorbance of the reaction mixture indicates higher reducing power of the sample. 

#### 2.2.3. Determination of Antioxidant Activity by Ferric Reducing Antioxidant Power (FRAP) Assay

FRAP assay was carried out according to Othman et al. [11] by monitoring the reduction of Fe^3+^-tripyridyl triazine (TPTZ) to blue colored Fe^2+^-TPTZ. The FRAP reagent was prepared by mixing 300 mM acetate buffer (pH 3.6), 10 mM TPTZ, and 20 mM ferric chloride in a ratio of 10 : 1 : 1. The reaction mixture containing 2 mL of FRAP reagent, 0.5 mL of extract, and 1 mL of distilled water was incubated for 10 min and the absorbance measured at 593 nm. Antioxidant potential of the sample was compared with the activity of 0.5 mL stock solution of 1 mg/mL FeSO_4_. 

#### 2.2.4. Determination of Ferrous Ion Scavenging (Metal Chelating) Activity

The chelating activity of the extract for ferrous ions was measured according to Zhao et al. [9]. The reaction mixture containing 0.5 mL of extract, 1.6 mL of deionized water, 0.05 mL of FeCl_2_ (2 mM) and 0.1 mL of ferrozine (5 mM) was incubated at 40°C for 10 min and the absorbance measured at 562 nm. The chelating activity was calculated as

(2) $$\text{Chelating}\;\text{rate}=\left[1-\frac{\left(A1\;-A2\right)}{\;A0}\right]\times 100,$$

where *A*0 represents the absorbance of the control (without extract) *A*1 represents the absorbance of reaction mixture, and *A*2 represents the absorbance without FeCl_2_. 

#### 2.2.5. Determination of Nitric Oxide (NO) Scavenging Activity

Nitric oxide production from sodium nitroprusside was measured according to Kang et al. [12]. An equal amount (6 mL) of sodium nitroprusside (5 mM) solution was mixed with 6 mL of extract and incubated at 25°C for 180 min. After every 30 min, 0.5 mL of the reaction mixture was mixed with an equal amount of Griess reagent (1% sulphanilamide, 2% phosphoric acid, and 0.1% napthylethylene diamine dihydrochloride), and absorbance was taken at 546 nm and compared with absorbance of 1 mg/mL of standard solution (sodium nitrite) treated in the same way with Griess reagent. 

#### 2.2.6. Determination of Total Phenolic Contents (TPC)

The total phenolic contents were determined colorimetrically using the Folin-Ciocalteau (FC) method according to Singleton et al. [13] with some modifications. Test sample (0.5 mL) was mixed with 0.2 mL of FC reagent and allowed to stand for 10 min to which 0.6 mL of 20% sodium carbonate was added and mixed completely. The reaction mixture was incubated at 40°C for 30 min. Absorbance of the reaction mixture was measured at 765 nm. Gallic acid was taken as standard. 

### 2.3. Optimization of Physiochemical and Nutritional Parameters

Different physiochemical and nutritional parameters were optimized for *Aspergillus fumigatus *to enhance their antioxidant potential. To see the effect of shaking on the antioxidant activity, the fungi were grown on Czapek dox's broth at shaking conditions at different RPM 100, 150, 200, and 250 at 25°C and compared with that of antioxidant potential under static conditions. Then, to monitor the antioxidant potential of fungi with respect to incubation period, the activity was monitored every 5th day up to 30 days of growth under static conditions. Further, the activity of *Aspergillus fumigatus *was checked in the culture broth obtained from the organisms grown at different temperatures (20°C, 25°C, 30°C, 35°C, 40°C, 45°C) and pH values (2 to 11). 

#### 2.3.1. Medium Optimization Using One-Factor-at-a-Time Classical Method

**(1) Screening of Different Carbon and Nitrogen Sources**

To find out the best carbon source, sucrose in the Czapek dox's medium was replaced with the same concentration of one of the sugars (glucose, maltose, lactose, starch, and glycerol) and to work out the best nitrogen source, NaNO_3_ in Czapek dox's medium was substituted with one or the other inorganic nitrogen source (KNO_3_, NH_4_NO_3_, NH_4_Cl, (NH_4_)_2_SO4, (NH_4_)HSO_4_) or nitrogen rich organic supplement (yeast extract, peptone, malt extract, urea, casein, soyabean meal).

#### 2.3.2. Statistical Optimization of the Medium

**(1) Plackett-Burman Experimental Design**

The Plackett-Burman experimental design is a valuable tool for the rapid evaluation of the effects of various medium components. Because this design is a preliminary optimization technique, which tests only two levels of each medium component, it cannot provide the optimal quantity of each component required in the medium. This technique, however, provides indications of how each component tends to affect the activity. The screening of most significant parameters affecting antioxidant potential was studied by the Plackett-Burman design. The 5 factors, which are components of Czapek dox's medium (sucrose, NaNO_3_, K_2_HPO_4,_ KCl, and MgSO_4_) were examined. Total 14 tests were designed including 12 combinations and 2 repetitions at central point which contain different concentration of each factor and the effect of each factor was determined by the difference between the average of the + and − responses. The significance level of effect of each factor was determined by student's *t* test. The most common mean of assessing significant value is the *P* value which was also evaluated for each factor.

**(2) Response Surface Methodology through Box-Behnken Designs**

On the basis of results from screening of different carbon and nitrogen sources through one-factor-at-a-time classical method and different components by Plackett-Burman design, sucrose and NaNO_3_ were found to be the best for antioxidant activity. Sucrose as carbon source, NaNO_3_ as nitrogen source and temperature were taken independent variables for the optimization by RSM using Box-Behnken designs of experiments. Each variable was studied at three levels (−1, 0, +1); for sucrose these were 5%, 3%, and 1%; NaNO_3_: 0.05%, 0.2%, and 0.35%; temperature: 15°C, 25°C and 35°C.

The experimental design included 17 flasks with five replicates having all the three variables at their central coded values. The DPPH assay, reducing power, ferrous ion scavenging activity, FRAP assay and, nitric oxide ion scavenging activity and their total phenolic contents was taken as responses *G*_(1–6)_. The mathematical relationship of response G (for each parameter) and independent variable *X* (*X*_1_, Sucrose; *X*_2_, NaNO_3_; and *X*_3_, temperature) was calculated by the following quadratic model equation:

(3) $$\begin{array}{cc} G_{\left(1\text{–}6\right)} & =\beta_{0}+\beta_{1}X_{1}+\beta_{2}X_{2}+\beta_{3}X_{3}+\beta_{11}X_{1}^{2}+\beta_{22}^{}X_{2}^{2}\\ & \;+\beta_{33}X_{3}^{2}+\beta_{12}X_{1}X_{2}+\beta_{13}X_{1}X_{3}+\beta_{23}X_{2}X_{3}, \end{array}$$

where *G* is the predicted response; *β*_0_, intercept; *β*_1_, *β*_2_, and *β*_3_, linear coefficients; *β*_11_, *β*_22_, and *β*_33_, squared coefficients and *β*_12_, *β*_13_, and *β*_23_ interaction coefficients. MINITAB version 11 statistical software was used to obtain optimal working conditions and generate response surface graphs. Statistical analysis of experimental data was also performed using this software.

### 2.4. Thermostability of Antioxidant Bioactivity

 To check the temperature sensitivity of the culture broth for antioxidant activity, it was subjected to 40°C, 60°C, 80°C, and 100°C for one h and the heat treated broth was then assayed for the residual antioxidant activity. 

### 2.5. Extraction with Different Organic Solvents

To work out the best organic solvent for extraction of bioactive component, the culture broth was treated with different solvents *viz* petroleum ether, chloroform, ethyl acetate, and butanol. Solvent extracted components were then evaporated to dryness in vaccuo and the resulting solids were reconstituted in methanol to get five times concentrated stock preparations which were then checked for their antioxidant potential by various assays. 

### 2.6. Toxicity Tests

The culture broth used to assess the antioxidant activity was subject to Ames test by using *Salmonella* reverse mutation based on histidine dependence and mutations in *S*.* typhimurium *[14]. Cytotoxicity was tested by using 3-(4,5-dimethylthiazol-2-yl)-2,5-diphenyl tetrazolium bromide (MTT) method. The fungal extracts (100 *μ*L) were incubated with 1 × 10^5^ RBCs/well in 96-well ELISA plates for 24 h. Then, 100 *μ*L MTT solution (0.5%, w/v) was added to each well and incubated further for 4 h. After incubation, the supernatant was removed and 100 *μ*L DMSO was added to each well to dissolve the formazan crystals. The absorbance was measured at 590 nm using an automated microplate reader. The wells with untreated cells served as control [15]. 

## 3. Results

### 3.1. Comparison of Antioxidant Potential By Different Quantitative Methods

The different assay procedures demonstrated *Aspergillus fumigatus* to possess potent antioxidant activity. The fungus showed a good scavenging effect of 69% on DPPH radicals. The reducing power (0.51) of culture broth was demonstrated by the reduction of Fe^3+^ to Fe^2+^. It also demonstrated effective ferric ion reduction based on FRAP assay and gave reduction rate of 45.2%. In addition, the chelation activity for ferrous ion was assayed and the fungal extracts chelated 48.3% of ferrous ion. The percentage rate of scavenging nitric oxide ion of fungal extracts was 50.2%. 

### 3.2. Total Phenolic Contents

The TPC of *Aspergillus fumigatus* extract have been expressed as gallic acid equivalent (GAE), that is, mg gallic acid/mL culture. The *Aspergillus fumigatus* possessed high TPC (5.68 mg/mL), which is positively correlated with their antioxidant potential. 

### 3.3. Antioxidant Activity under Different Physiochemical Conditions

#### 3.3.1. Effect of Shaking Conditions

The experiments carried out to see the effect of shaking at different RPM demonstrated static culture to give better antioxidant yield in comparison to shake flask cultures, which resulted in steady decline in the activity with increase in RPM. The scavenging effect was 69% under static culture conditions while it was 62.3% on shake flask culture. Again, reducing potential was 0.51 under static conditions and 0.42 at shaking conditions. Ferric ion reduction was also more in static (45.2%) than in shake cultures (40.1%). Ferrous and nitric ion scavenging activity decreased under shaking condition and it was 48.3% and 50.2% under static conditions, respectively. TPC value was also high in static culture (5.68 mg/mL) as compared to shake culture (4.2 mg/mL). Thus, further optimization was carried out under static conditions. 

#### 3.3.2. Effect of Growth Period

The antioxidant potential as assayed by different procedures was best expressed on the 10th day with the scavenging effect of 68.8%, 48.3%, and 50.2% on DPPH, ferrous and NO ion, respectively, and 45.2% for ferric ion reduction. The antioxidant activity as assayed by various procedures and TPC remained more or less similar on 15th day and subsequently declined upto 30 days. The decline in the TPC correlated uniformly with the decline in the activity. 

#### 3.3.3. Effect of Temperature and pH

The antioxidant potential was best observed at 25°C and in between pH 5–7 with the scavenging effect of 69%, 48.8%, and 50.7% on DPPH, ferrous; NO ion, respectively, and 45.1% for ferric ion reduction. There was no significant loss in the antioxidant activity or TPC up to 35°C while activity decreased at 40°C. Neither any antioxidant activity nor TPC could be detected at extreme pH values (2, 3, 10, 11, and 12) by any of the methods as there was no fungal growth at these extreme conditions. 

#### 3.3.4. Effect of Different Carbon and Nitrogen Sources on Antioxidant Potential

Initially, to assess the antioxidant potential by various assay procedures, all the experimentation was done by growing *Aspergillus fumigatus *on Czapek dox's broth medium. In order to find the optimal carbon source, sucrose was replaced with different sugars. However, sucrose remained the best to support the maximum antioxidant activity and the order followed was sucrose > dextrose > maltose > starch > lactose > glycerol. Sucrose was thus selected as carbon source for further experimentation (Table 1).

Similarly, NaNO_3_ turned out to be the best nitrogen source to support maximum antioxidant potential. Peptone and yeast extract were also good sources of nitrogen, while urea gave the poorest activity (Table 2). The antioxidant profile of *Aspergillus fumigatus *for different nitrogen sources remained the same irrespective of assay procedure adopted. NO ion scavenging activity was monitored up to 180 min which increased gradually with respect to time. However, data pertaining to 180 min is only shown.

The highest TPC yield was 5.68 mg/mL in the presence of sucrose and NaNO_3_ in the medium. On the basis of the above results, Czapek dox's broth medium was chosen for the remaining experiments. 

### 3.4. Statistical Optimization of the Medium

#### 3.4.1. Plackett-Burman Design for Selection of Significant Components

A Plackett-Burman design experiment was employed to evaluate the influence of five factors (sucrose, NaNO_3_, K_2_HPO_4,_ KCl, and MgSO_4_) and their importance in culture medium to obtain better antioxidant activity. Antioxidant potential of *Aspergillus fumigatus *assayed by different procedures and extracellularly produced total phenolic content that varied significantly with the 14 run of different combinations of the media components (Table 3). The maximum antioxidant potential along with high TPC was observed in run order 13 and run order 14 which was followed by run order 5. The results were subjected to regression analysis and the analysis of variance (ANOVA) which revealed sucrose and NaNO_3_ to have statistically significant effect on antioxidant potential with *P* value ≤.05 and ≤.5, respectively, thus showing that of the five variables, only sucrose and NaNO_3_ played a critical role for antioxidant activity. Based on these results, sucrose and NaNO_3_ were selected as two variables to optimize the medium composition by RSM. To know the optimum temperature and its interaction with other variables (sucrose and NaNO_3_), it was chosen as a third variable as it is an important physical parameter that affects the activity as well as fungal growth. 

#### 3.4.2. Box-Behnken Design

**(1) Fitting the Model**

The data obtained from quadratic model equation was found to be significant. It was verified by *F* value and the analysis of variance (ANOVA) by fitting the data of all independent observations in response surface quadratic model. The results for model *F*-value implies that the model is significant which indicates it to be suitable to represent adequately the real relationship among the parameters used. *R* ^2^ value for all the responses ranged between 82 to 87%, which showed suitable fitting of the model in the designed experiments (Table 4). The final predictive equations for each response: DPPH assay (*G*_1_), reducing power (*G*_2_), ferrous ion scavenging activity (*G*_3_), FRAP assay (*G*_4_), and nitric oxide ion scavenging activity (*G*_5_) and their total phenolic contents (*G*_6_) obtained are as follow:

(4) $$\begin{array}{cc} & G_{\left(1\right)}\\ & =12.19+22.55X_{1}+93.48X_{2}+0.37X_{3}-2.38X_{1}^{2}+{132.56X}_{2}^{2}\\ & \;-{0.0X}_{3}^{2}-51.08X_{1}X_{2}+0.08X_{1}X_{3}-0.17X_{2}X_{3},\\ & G_{\left(2\right)}\\ & =0.647+0.104X_{1}+0.65X_{2}-0.033X_{3}-0.006X_{1}^{2}+2.78X_{2}^{2}\\ & \;-{0.0006X}_{3}^{2}-0.66X_{1}X_{2}+0.0033X_{1}X_{3}+0.005X_{2}X_{3},\\ & G_{\left(3\right)}\\ & =3.69+14.24X_{1}+78.73X_{2}+0.60X_{3}-{1.22X}_{1}^{2}+{185.67X}_{2}^{2}\\ & \;-{0.0X}_{3}^{2}-50.17X_{1}X_{2}+0.13X_{1}X_{3}-0.42X_{2}X_{3},\\ & G_{\left(4\right)}\\ & =5.02+14.48X_{1}+74.86X_{2}+0.32X_{3}-1.12X_{1}^{2}+222.4X_{2}^{2}\\ & \;+0.0X_{3}^{2}-54.17X_{1}X_{2}+0.12X_{1}X_{3}-0.35X_{2}X_{3},\\ & G_{\left(5\right)}\\ & =5.58+14.20X_{1}+75.93X_{2}+0.69X_{3}-1.18X_{1}^{2}+201.0X_{2}^{2}\\ & \;-0.0X_{3}^{2}-51.17X_{1}X_{2}+0.13X_{1}X_{3}-0.50X_{2}X_{3},\\ & G_{\left(6\right)}\\ & =-1.76+0.84X_{1}+17.38X_{2}+0.23X_{3}+0.09X_{1}^{2}+41.56X_{2}^{2}\\ & \;-0.0X_{3}^{2}-11.42X_{1}X_{2}+0.04X_{1}X_{3}-0.05X_{2}X_{3}. \end{array}$$

The optimized values of factors were validated by repeating the experiment in triplicate flasks.

**(2) Effect of Different Variables on DPPH Assay**

**Figure 1:**
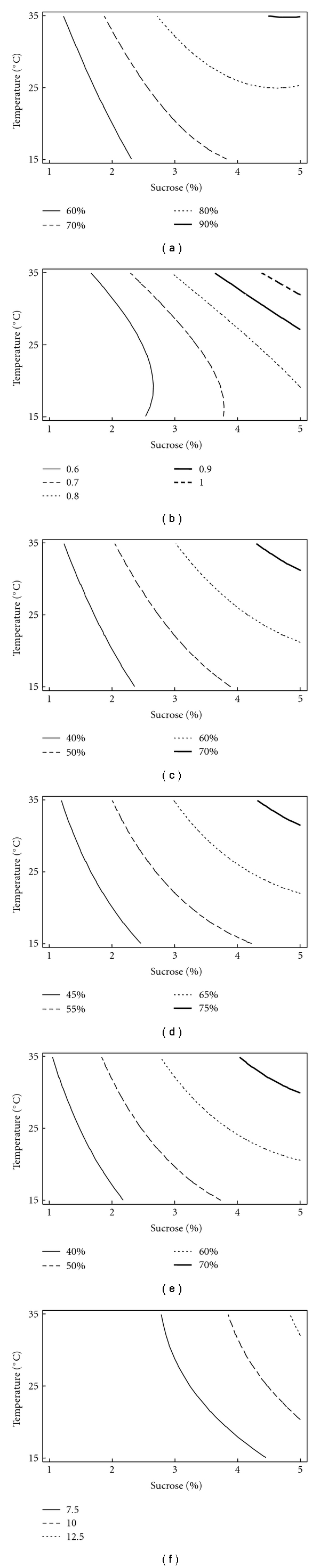
Contour graph showing effect of different variables on antioxidant potential (% activity) as assayed by different procedures (a) 1,1-diphenyl-2-picryl hydrazyl free radical (DPPH) assay (hold value: 0.05% of sodium nitrate); (b) reducing power (in absorbance) (hold value: 0.05% of sodium nitrate); (c) ferrous ion scavenging activity (hold value: 0.05% of sodium nitrate); (d) nitric oxide ion scavenging activity (hold value: 0.05% of sodium nitrate); (e) FRAP assay (hold value: 0.05% of sodium nitrate); (f) total phenolic content (mg/mL) (hold value: 0.05% of sodium nitrate).

Sucrose significantly affected the DPPH activity. The linear effect (*X*_1_) and the squared effect (*X*_1_^2^) were significant (*P* value <.05), and the interactive effect (*X*_1_ *X*_2_) was highly significant (*P* value <.005). The response surface graphs showed the highest activity at 3–5% sucrose but with the least amount of NaNO_3_ while the activity decreased with the decrease in sucrose concentration and with the increase in the concentration of NaNO_3_ at a constant temperature of 25°C. Maximum DPPH scavenging effect (90%) was obtained at 5% sucrose, 0.05% NaNO_3_, and at 35°C (Figure 1(a)).

**(3) Effect of Different Variables on Reducing Power**

Linear effects (*X*_1_, *X*_3_), squared effects (*X*_2_^2^, *X*_3_^2^), and interactive effect between sucrose and temperature (*X*_1_ *X*_3_) was significant with *P* value <.5. Interactive effect (*X*_1_ *X*_2_) was most significant at *P* value ≤.005. The response surface graphs showed the highest reducing potential with an absorbance of 1.0 at 5% sucrose with 0.05% of NaNO_3_ and at a temperature of 35°C (Figure 1(b)).

**(4) Effect of Different Variables on FRAP Assay, Ferrous Ion, and Nitric Oxide Ion Scavenging Activity**

Effect of variables was similar on FRAP assay, ferrous ion, and nitric oxide ion scavenging activity. Interactive effect (*X*_1_ *X*_2_) was most significant with *P* value ≤.005. While linear (*X*_2_), squared effect (*X*_1_^2^, *X*_2_^2^) and interactive effect (*X*_1_ *X*_3_) showed significance at *P* ≤ .5. At 35°C, with medium composition of 5% sucrose and 0.05% of NaNO_3_, ferric reducing antioxidant power was highest (70%) as compared to other medium conditions (Figure 1(c)). Similarly, the highest scavenging effect of 75% for nitric oxide ion was observed at 35°C with 5% and 0.05% of sucrose and NaNO_3_, respectively, (Figure 1(d)). The chelating effect (70%) was highest at 35°C in the medium containing 5% sucrose with 0.05% NaNO_3_. Antioxidant potential as assayed by different procedures demonstrated decrease in activity with increase of NaNO_3_ concentration and decrease in the temperature and sucrose concentration (Figure 1(e)).

**(5) Effect of Different Variables on Total Phenolic Content**

The interactive effect (*X*_1_ *X*_2_) was highly significant with *P* value ≤.005 while linear (*X*_2_) and squared effect of sodium nitrate (*X*_2_^2^), and interactive effect between sucrose-temperature (*X*_1_ *X*_3_) is significant with *P* value ≤.5. The highest amount of TPC was obtained at 5% sucrose and 0.05% NaNO_3_ concentration at 35°C (Figure 1(f)), and yield decreased with the decrease in temperature and sucrose concentration and with increase in NaNO_3_ concentration.

**(6) Validation of Results**

Thus from the overall assessment, 5% sucrose, 0.05% NaNO_3_, and incubation temperature of 35°C and retaining other media components at standard concentration in Czapek Dox's medium may be regarded as the optimized conditions for different assay procedures. The *F* value and *R* ^2^ value showed that the model correlated well with measured data and was statistically significant. To confirm the adequacy of the model for predicting maximum scavenging activity, the verification experiments using the optimum medium composition, as described above, were carried out in triplicates which showed 89.8%, 70.1%, and 74.2% scavenging effect for DPPH radical, ferrous ion and nitric oxide ion, respectively. The yield for TPC was 12.3 mg/mL and reducing power showed 1.0 absorbance with 70.5% activity for FRAP assay. A good agreement between the predicted and experimental results verified the validity of the model and the improvement of antioxidant activity indicated that RSM is a powerful tool for determining the exact optimal values of the individual factors and the maximum response value.

### 3.5. Thermostability of Antioxidant Activity

The culture filtrate of *Aspergillus fumigatus *showing antioxidant activity was found to be relatively thermostable as it suffered a slight loss in its activity with increase in temperature. At 40°C, the activity decreased by only 7% in fungal extracts, while at 100°C it suffered a maximum loss of 50% in its activity. 

### 3.6. Extraction of Bioactive Compound in Different Organic Solvents

The extraction of culture broth with different organic solvents revealed ethyl acetate to be the best to elute the components responsible for antioxidant potential and it was followed by chloroform and butanol. Petroleum ether extracts did not show any activity. The ethyl acetate extract showed 74.6%, 68.8%, and 70.5% scavenging activity for DPPH, ferrous, and NO ion, respectively. Ferric reducing antioxidant power and reducing potential was 65.6% and 1.1. The chloroform extract showed 0.78 reducing potential with ferric ion reduction of 55.3% and 67.4%, 56.7%, and 56.9% of scavenging effect for DPPH, ferrous, and NO ion, respectively. Butanol extract exhibited reducing potential of 0.34 and 38.7% of ferric reducing antioxidant power with scavenging effect of 50%, 35.5%, and 40.8% for DPPH, ferrous and NO ion, respectively. 

### 3.7. Toxicity Tests

The cell-free fungal extracts, when studied for Ames test, showed no mutagenic activity as no bacterial colony was observed on agar plates containing fungal extracts, while more than 1000 colonies were observed on positive control (sodium azide) containing plate. Similarly, results obtained from MTT assay revealed that the cell-free extracts were noncytotoxic and showed much higher absorbance (0.775) as compared to positive control (0.107). 

## 4. Discussion

A number of fungi, in particular mushrooms, have been known to possess good antioxidant activity [16]. However, much work still needs to be done to explore filamentous fungi for antioxidant activity, and the results shown by *Aspergillus fumigatus* support this contention [17]. Antioxidant activity, as assayed by different methods, demonstrated static culture of *Aspergillus fumigatus *to be more suitable as compared to shake flask culture. This supports the earlier observation of various researchers who have used static conditions [18] or low RPM (between 100 to 150 RPM) [17, 19, 20]. It might be attributed to a low amount of phenolic compounds produced under shaking conditions, which have been held responsible for antioxidant activity of fungi. Ten days of incubation period was optimum for antioxidant activity, and the subsequent decline in bioactivity could be due to the exhaustion of nutrients available for the fungi. This decline may also be attributed to the degradation of secondary metabolites (phenolic compounds) already produced by fungi as supported by decline in the phenolic content.

The comparison of antioxidant activity of the cell-free culture broth obtained from the fungus grown at different temperatures revealed 25°C to be the optimum temperature, which correlate positively with its phenolic content. No activity was detected at pH extremes, which was optimally best between the pH 5 and 7. The present results corroborate the previous studies done by Miao et al. [21] on antibacterial activity from fungal sources in which there was no bioactivity at pH extremes. This may be due to delayed metabolite production caused by delayed mycelial growth or due to reduced production of bioactive metabolites under such pH conditions. This shows that pH of the growth medium can also significantly affect the production of secondary metabolites. The pH is related to permeability characteristics of the cell wall and membrane, thus affecting either ion uptake or loss to the nutrient medium [22].

Sucrose and sodium nitrate were found to be the most promising carbon and nitrogen sources for obtaining the best antioxidant activity by* Aspergillus fumigatus *which is in consonance with earlier studies carried out on *Aspergillus candidus* [23]. However, it contravenes the general perception that glucose and starch are the best carbon source for fungal growth. The results thus suggest that a fungal species may have the ability to utilize a particular carbon source for vegetative growth but may not be able to use it for production of specialized metabolites. This signifies that availability of easily utilizable carbon and nitrogen sources (sucrose and NaNO_3_) may lead to the formation of secondary metabolites. All carbon and nitrogen sources are divided into quickly metabolizable sources and sustainable sources. Quickly metabolizable sources are beneficial for faster growth of microorganisms and relieving their need for long-term accumulation of products. Sucrose and NaNO_3_ are regarded as sustainable sources, which favors the production of secondary metabolites as these are the products of later growth [24]. The study thus demonstrated the basic composition of Czapek Dox's medium to be the best for effective production of antioxidant activity. In fact, culture media designing has a major impact on the growth of microbes and the production of microbial products [21].

Further analysis of the effect of the medium constituents through Plackett Burman design showed sucrose and NaNO_3_ to be significant but the significance of NaNO_3_ was less than 50%. The results got further support from the RSM observations where low concentration of NaNO_3_ in the medium favors the antioxidant activity. This demonstrates importance of nitrogen sources in regulating the production of secondary metabolites [25]. Sucrose is beneficial for the growth of fungi as well as for production of secondary metabolites which are responsible for their antioxidant activity.

Though KCl, MgSO_4_, and K_2_HPO_4_ did not significantly affect the antioxidant activity, still they are retained are retained at standard concentration in Czapek Dox's medium because magnesium and potassium are required by all the fungi for a variety of regulatory functions and control the biosynthesis of various secondary metabolites. This shows that the medium most suitable for growth may or may not be equally effective for secondary metabolites and thus enhancement of secondary metabolites can only be achieved through systematic manipulation of different parameters [26].

Thermostability studies on cell-free extract demonstrated that metabolites responsible for antioxidant activity are quite stable at 40°C. Of the different organic solvents tried for extraction, ethyl acetate showed the best activity followed by chloroform and butanol extract. Our observations with ethyl acetate extracts are in consonance with earlier studies [23, 27]. Further, the results of ethyl acetate extracts were quite comparable with the activity of ascorbic acid (96.7%), BHA (95.1%), and alpha tocopherol (94.7%).

It is commonly known that the antioxidative effects are mainly due to redox properties of phenolic compounds which can play an important role in absorbing and neutralizing the free radicals by acting as reducing agents and hydrogen donor, quenching singlet and triplet oxygen or decomposing peroxides [19]. The importance of phenolic contents has been endorsed by their high content in *Aspergillus fumigatus *and their antioxidant activity is quite comparable to that of many mushrooms as well as medicinal plants. Further, the better production of phenolics under optimized conditions also enhanced the antioxidant activity. 

 The results obtained indicate *Aspergillus fumigatus *to be a potent antioxidant producer having broad spectrum against various free radicals. Previous studies have shown the linear correlation between total phenolic content and antioxidant activity; total phenolic content of *Aspergillus fumigatus *correlated well with the antioxidant activity which is in consonance with earlier studies [28]. The extract obtained from *Aspergillus fumigatus *showed good activity against DPPH radical by neutralizing the free radical character of purple color DPPH, either by transfer of electron or hydrogen atom to yellow-colored diamagnetic molecule revealing hydrogen donating property of phenolic compounds present in the extract which can be supported by the positive correlation (*r* = 0.817) between the results of DPPH assay and TPC [29]. Similarly, positive correlation (*r* = 0.815) was found between reducing power assay and TPC. Reducing power assay proves the potential of the phenolic compounds in the extracts to act as reductones that inhibit lipid peroxidation by donating a hydrogen atom thereby terminating the free radical chain reaction. Moreover, this reducing potential may be due to the di- or monohydroxy substitution in the aromatic rings that possess potent hydrogen donating ability [9]. Results of FRAP assay are also positively correlated (*r* = 0.856) with TPC and good activity of the fungal extract for FRAP assay denotes its reducing potential. Generally, the reducing properties are associated to the breaking of free radical chain by donating a hydrogen atom [11]. The extracts also showed appreciable chelating activity of metals, as the transition metals such as ferrous ion can stimulate lipid peroxidation by generating hydroxyl radicals through Fenton reaction. The chelating activity for ferrous ion was assayed by the inhibition of formation of red-colored ferrozine and ferrous complex. There was positive-correlation (*r* = 0.819) between chelating activity and TPC [9]. As evident from studies, the cell-free extracts are able to scavenge nitric oxide ion, and correlation with TPC was found to be positive (*r* = 0.813). 

Most of the literature is available on antioxidant activity of plants and mushrooms, though some of the fungi are known to produce antioxidant activity. To the best of our knowledge, apparently this is the first systematic report on antioxidant activity of *Aspergillus fumigatus* demonstrated by different assay procedures and its optimization by statistical methods. Under optimal condition, *Aspergillus fumigatus *showed 89.8%, 70.1%, and 74.2% scavenging effect for DPPH radical, ferrous ion and nitric oxide ion, respectively. The yield for TPC was 12.3 mg/mL and reducing power showed absorbance of 1.0 and 70.5% activity for FRAP assay. The results showed the scavenging effect for DPPH radical, ferrous ion, and nitric oxide ion was enhanced by 1.3, 1.4, and 1.4 folds, respectively, while reducing potential and ferric reduction rate was enhanced by 2.0 and 1.3 folds. The production of TPC was enhanced by 2.1 folds. These results are also comparable with the antioxidant activity of various other fungi, *Aspergillus candidus*, *Chaetomium* sp.,* Cladosporium* sp,* Colletotrichum gloeosporioides* [30] and many mushrooms such as *Lentinus edodes*, *Volvariella volvacea* [16] and many medicinal plants like *Amaranthus paniculatus, Aerva lanata, Coccinia indica*,* Coriandrum sativum *[31]. To further highlight the importance of the study, the results of the cell-free extract also exhibited higher activity than synthetic antioxidants (BHA and BHT). 

## 5. Conclusions

Hence, the above study suggests that not only mushrooms and plants but some other fungi may also be a good source of antioxidant compounds and *Aspergillus fumigatus *is one such potential candidate offering a better scope for production and easier downstreaming of such bioactive compounds as toxicity studies proved that extract is neither cytotoxic nor mutagenic. These findings will facilitate the further studies to gain better understanding of production of bioactive metabolites in fungi, which will be helpful in their biotechnological mass production in the near future. 

## Figures and Tables

**Table 1:** Effect of various carbon sources on antioxidant potential of *Aspergillus fumigatus. *

% activity	Dextrose	Maltose	Lactose	Starch	Glycerol	Sucrose
DPPH* assay	62.2 ± 0.06	60.8 ± 0.5	50.6 ± 0.4	56.7 ± 0.4	32.9 ± 0.2	68.96 ± 0.47
Reducing power	0.42 ± 0.03	0.40 ± 0.02	0.3 ± 0.02	0.4 ± 0.7	0.16 ± 0.4	0.510 ± 00
Fe^2+^ scavenging activity	45.3 ± 0.9	42.1 ± 0.08	35.3 ± 0.1	40.3 ± 0.55	24.3 ± 0.5	48.3 ± 0.3
FRAP** assay	40.7 ± 0.2	38.8 ± 0.09	32.9 ± 0.2	35.4 ± 0.3	25.6 ± 0.5	45.2 ± 0.2

NO*** scavenging activity						

30 min	24.6 ± 0.2	23.8 ± 0.1	12.5 ± 0.4	16.7 ± 0.01	10.3 ± 0.2	28.2 ± 0.7
60 min	30.2 ± 0.3	30.5 ± 0.4	18.4 ± 0.02	22.6 ± 0.3	18.4 ± 0.5	35.2 ± 0.02
90 min	38.3 ± 0.1	34.2 ± 0.6	21.8 ± 0.2	26.8 ± 0.02	20.4 ± 0.8	40.2 ± 0.8
120 min	40.3 ± 0.1	38.6 ± 0.1	26.3 ± 0.2	30.2 ± 0.4	25.5 ± 0.3	45.3 ± 0.11
180 min	45.3 ± 0.02	40.8 ± 0.02	30.8 ± 0.5	35.2 ± 0.88	28.9 ± 0.32	50.2 ± 0.45
TPC**** (mg/mL)	4.2 ± 0.1	3.8 ± 0.5	2.1 ± 0.07	3.2 ± 0.4	1.0 ± 0.7	5.68 ± 0.03
Biomass (mg)	35.4 ± 0.2	30.4 ± 0.04	20.6 ± 0.06	26.7 ± 0.3	12.6 ± 0.02	40.8 ± 0.3

*DPPH: 1.1-diphenyl-2-picryl hydrazyl; **FRAP: ferric reducing antioxidant power; ***NO: nitric oxide; ****TPC: total phenolic content; values are means of three replicates ± S.D.

**Table 2:** Effect of various nitrogen sources on antioxidant potential of *Aspergillus fumigatus. *

Antioxidant activity (%)							
Nitrogen sources	DPPH* assay	Reducing power	Fe^2+^ scavenging activity	FRAP** assay	NO*** scavenging activity	TPC**** (mg/mL)	Biomass (mg)
Nitrogen rich organic supplements							

Yeast extract	65.8 ± 0.6	0.48 ± 0.7	42.7 ± 0.1	40.2 ± 0.3	30.7 ± 0.04	34.8 ± 0.3	38.8 ± 0.04
Peptone	65.8 ± 0.5	0.463 ± 0.6	42.3 ± 0.2	40.1 ± 0.2	30.1 ± 0.6	35.2 ± 0.2	37.6 ± 0.06
Malt extract	58.3 ± 0.5	0.35 ± 0.3	37.8 ± 0.5	35.6 ± 0.01	25.7 ± 0.2	30.5 ± 0.3	35.8 ± 0.5
Casein	60.3 ± 0.07	0.40 ± 0.07	40.5 ± 0.4	37.7 ± 0.6	22.8 ± 0.4	31.9 ± 0.5	35.7 ± 0.2
Soyabean meal	52.3 ± 0.5	0.22 ± 0.02	35.2 ± 0.5	32.7 ± 0.02	20.7 ± 0.02	25.4 ± 0.1	28.7 ± 0.5
Urea	20.4 ± 0.1	—	—	—	—	—	8.6 ± 0.04

Inorganic nitrogen sources							

KNO_3_	45.2 ± 0.05	0.302 ± 0.01	28.5 ± 0.6	25.7 ± 0.03	15.8 ± 0.05	17.8 ± 0.04	20.6 ± 0.78
(NH_4_)_2_SO_4_	46.7 ± 0.03	0.320 ± 0.5	28.9 ± 0.5	26.7 ± 0.5	15.3 ± 0.1	28.9 ± 0.1	20.7 ± 0.6
(NH_4_)HSO_4_	40.3 ± 0.06	0.206 ± 0.1	25.3 ± 0.03	22.8 ± 0.7	10.3 ± 0.2	18.9 ± 0.1	20.8 ± 0.3
NH_4_NO_3_	36.2 ± 0.01	0.18 ± 0.03	24.5 ± 0.1	21.9 ± 0.8	10.8 ± 0.5	12.8 ± 0.2	18.6 ± 0.4
NaNO_3_	68.9 ± 0.4	0.510 ± 00	48.3 ± 0.3	45.2 ± 0.22	28.2 ± 0.7	35.2 ± 0.02	40.2 ± 0.8
NH_4_Cl	50.3 ± 0.7	0.26 ± 0.5	30.2 ± 0.1	28.6 ± 0.3	16.9 ± 0.2	20.6 ± 0.3	25.7 ± 0.1

*DPPH: 1.1-diphenyl: 2-picryl hydrazyl; **FRAP: ferric reducing antioxidant power; ***NO: nitric oxide; ****TPC: total phenolic content; values are means of three replicates ± S.D.

**Table 3:** Plackett-Burman design variables with antioxidant potential of *Aspergillus fumigates. *

	Variables (%)					Antioxidant activity (%)					
Run	Sucrose	NaNO_3_	K_2_HPO_4_	MgSO_4_	KCl	DPPH* assay	Reducing power	Fe^2+^scavenging activity	FRAP** assay	NO*** scavenging activity	TPC**** (mg/mL)
1	5.0	0.000	0.18	0.000	0.000	58.1	0.48	44.3	45.7	48.30	4.80
2	5.0	0.350	0.00	0.090	0.000	65.4	0.72	48.1	50.3	52.30	5.80
3	0.0	0.350	0.18	0.000	0.090	25.3	0.20	20.3	25.6	28.20	1.80
4	5.0	0.000	0.18	0.090	0.000	50.3	0.40	40.9	42.1	45.30	4.80
5	5.0	0.350	0.00	0.090	0.090	72.1	0.63	54.2	56.3	60.20	6.20
6	5.0	0.350	0.18	0.000	0.090	65.4	0.55	48.3	50.6	55.60	5.30
7	0.0	0.350	0.18	0.090	0.000	23.4	0.19	18.6	20.3	25.40	1.40
8	0.0	0.000	0.18	0.090	0.090	48.2	0.36	37.3	40.1	45.20	4.00
9	0.0	0.000	0.00	0.090	0.090	20.8	0.11	15.2	17.2	20.20	1.01
10	5.0	0.000	0.00	0.000	0.090	60.3	0.50	46.2	45.3	48.30	5.00
11	0.0	0.350	0.00	0.000	0.000	54.3	0.45	42.1	40.2	45.03	4.50
12	0.0	0.000	0.00	0.000	0.000	0.0	0.00	0.0	0.0	0.00	0.00
13	2.5	0.175	0.09	0.045	0.045	69.2	0.58	49.0	47.2	52.20	6.00
14	2.5	0.175	0.09	0.045	0.045	68.4	0.57	48.2	47.1	52.10	6.10

*DPPH: 1.1-diphenyl -2-picryl hydrazyl; **FRAP: Ferric reducing antioxidant power; ***NO: nitric oxide; ****TPC: total phenolic content.

**Table 4:** Box-Behnken designs of different variables with antioxidant potential of *Aspergillus fumigates. *

Variables (%)				Antioxidant activity (%)					
Run	Sucrose	NaNO_3_	Temperature	DPPH Assay	Reducing power	Fe^2+^ scavenging activity	FRAP assay	NO scavenging activity	TPC (mg/mL)
1	1	0.05	25	52.3	0.42	38.4	35.3	41.8	4.6
2	5	0.05	25	77.8	0.81	62.3	60.2	65.2	10.3
3	1	0.35	25	78.1	0.83	64.4	65.4	68.1	10.9
4	5	0.35	25	42.3	0.42	28.1	25.3	30.1	2.9
5	1	0.20	15	45.4	0.48	30.4	28.2	32.4	3.1
6	5	0.20	15	47.8	0.48	32.3	30.3	35.2	3.8
7	1	0.20	35	69.2	0.63	50.1	48.3	52.4	6.1
8	5	0.20	35	78.0	0.89	62.4	60.1	65.3	10.2
9	3	0.05	15	70.5	0.71	50.2	50.2	53.2	6.2
10	3	0.35	15	69.3	0.62	50.8	49.2	53.3	6.0
11	3	0.05	35	76.4	0.78	56.2	54.3	59.3	6.9
12	3	0.35	35	74.2	0.72	54.3	51.2	56.4	6.4
13	3	0.20	25	69.0	0.59	50.1	45.3	52.6	5.8
14	3	0.20	25	68.8	0.61	50.2	46.3	52.1	5.8
15	3	0.20	25	69.3	0.58	48.3	46.2	50.1	6.1
16	3	0.20	25	69.8	0.62	48.2	47.2	52.4	6.0
17	3	0.20	25	68.9	0.51	48.3	45.2	50.2	5.7
